# Experimental Investigation of the Cd-Pr Phase Diagram

**DOI:** 10.1371/journal.pone.0094025

**Published:** 2014-04-09

**Authors:** Thomas L. Reichmann, Herta S. Effenberger, Herbert Ipser

**Affiliations:** 1 Department of Inorganic Chemistry (Materials Chemistry), University of Vienna, Wien, Austria; 2 Department of Mineralogy and Crystallography, University of Vienna, Wien, Austria; Griffith University, Australia

## Abstract

The complete Cd-Pr equilibrium phase diagram was investigated with a combination of powder-XRD, SEM and DTA. All intermetallic compounds within this system, already reported in literature, could be confirmed: CdPr, Cd_2_Pr, Cd_3_Pr, Cd_45_Pr_11_, Cd_58_Pr_13_, Cd_6_Pr and Cd_11_Pr. The corresponding phase boundaries were determined at distinct temperatures. The homogeneity range of the high-temperature allotropic modification of Pr could be determined precisely and a limited solubility of 22.1 at.% Cd was derived. Additionally, single-crystal X-ray diffraction was employed to investigate structural details of Cd_2_Pr; it is isotypic to the AlB_2_-type structure with a *z* value of the Cd site of 0.5. DTA results of alloys located in the adjacent two-phase fields of Cd_2_Pr suggested a phase transformation between 893 and 930°C. For the phase Cd_3_Pr it was found that the lattice parameter *a* changes linearly with increasing Cd content, following Vegard’s rule. The corresponding defect mechanism could be evaluated from structural data collected with single-crystal XRD. Introduction of a significant amount of vacancies on the Pr site and the reduction in symmetry of one Cd position (8*c* to 32*f*) resulted in a noticeable decrease of all R-values.

## Introduction

An ever increasing demand of energy, especially in developing countries with high economic growth, makes the utilization of nuclear energy sometimes inevitable. On the back-end of the nuclear fuel cycle an immense amount of nuclear waste is being produced which has to be overcome. Indeed, low-level and intermediate-level radioactive waste is currently stored in interim storage facilities or deposited in geological repositories. Solutions for high-level waste are currently still in a planning stage and thus this waste is actually stored on-site. For an efficient utilization of nuclear fuels as well as to decimate the amount and radioactivity of nuclear waste, reprocessing techniques have been established.

Focusing onto reprocessing of nuclear waste, this is currently practiced by means of solvent extraction of actinides. Aqueous extraction of radioactive waste is suffers from several problems, described repeatedly in literature [1]–[5]. To overcome the major disadvantages of these hydro-metallurgical reprocessing methods, electro-transport and reductive extraction is applied to separate actinides and lanthanides from high-level radioactive waste. In this type of non-aqueous method liquid Cd is used to extract minor actinides from a liquid chloride salt solvent. The procedure is described in detail in [6]–[8].

The extraction behaviour of different elements between a molten chloride salt phase and a liquid metal strongly depends on the standard free energy of formation of the corresponding chlorides as well as on the activity coefficients of the extracted elements in the respective intermetallic compounds. Thus the separation factors are strongly influenced by the employed liquid metal which is preferentially Cd [9]. Therefore, a detailed knowledge of the respective Cd-RE phase diagrams as well as of the thermodynamic stabilities of the corresponding intermetallic compounds is of great importance. This was the reason for initiating a series of thermodynamic and phase diagram studies of different Cd-RE systems (cf. Refs. [10]–[11]).

Concerning the system Cd-Pr, only limited information about the phase diagram is available from literature. A compilation of structural data of the respective intermetallic compounds together with a partial phase diagram, determined by Johnson et al. [12], is given by Gschneidner and Calderwood [13]. Johnson et al. reported liquidus data in the composition range up to 1.83 at.% Pr, determined by chemical analysis of filtered samples of the corresponding equilibrium phases. The latter authors further applied differential thermal analysis and presented at least two invariant reactions. They argued for a degenerate eutectic reaction between Cd and Cd_11_Pr and a peritectic decomposition of Cd_11_Pr at 570°C, at which temperature 3.5 at.% Pr are soluble in liquid Cd. In a previous work Veleckis and Van Deventer [14] determined experimentally an invariant reaction temperature for a eutectic reaction between Pr and CdPr at 435°C.

On the basis of all available thermodynamic and phase diagram data Kurata and Sakamura [15] made a CALPHAD-type optimization and presented a partial Cd-Pr phase diagram between 0–25 at.% Pr.

Although the information on phase equilibria and invariant reactions in the Cd-Pr system is limited, an extensive survey of the occurring intermetallic compounds is given in literature. Table 1 compiles crystallographic data for the binary compounds together with their homogeneity ranges according to Reichmann and Ipser [11]. Related phase diagrams between Cd and lanthanides indicate that numerous intermetallic compounds are isotypic or even isostructural. With the exception of the results of vapour pressure measurements given in Refs. [10], [11], no significant homogeneity ranges of these phases are known. As mentioned in Table 1 some solubility was determined for at least five compounds whereas Cd_6_Pr and Cd_11_Pr were described as line compounds. Noticeably, all compounds are located within the composition range 50–92 at.% Cd. The compound richest in Pr, CdPr, adopts the CsCl-structure type (*B*2). It was determined with energy-dispersive X-ray spectroscopy that CdPr dissolves around 3 at.% Pr whereas no significant solubility of Cd was found (cf. Table 1). The next compound richer in Cd is Cd_2_Pr (structure type *C*6). Compounds with the respective composition ratios 1∶1 and 2∶1 were found in all phase diagrams between Cd and lanthanides. Compounds with the general formula Cd_2_Ln, where Ln stands for lanthanides, were investigated extensively. They crystallize either in space-group *P*6/*mmm* or *P
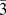
m*1 [19]–[24]. The only exception is Cd_2_Eu for which space group *Imma* was found [25]. Compounds with space-group symmetry *P*6/*mmm* adopt the structure type AlB_2_ (Pearson code *hP*3) with Wyckoff sequence *d a*; the site positions are (⅓⅔½) and (000). The compounds with space-group *P
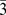
m*1 maintain the Pearson code and Wyckoff sequence, the site positions are (⅓⅔ *z*) and (000). However, the *z* parameter of the site 2(*d*) varies either between 0.042 and 0.080 or between 0.42 and 0.43. Phases adopting *z* parameters around 0.42 can be considered as having a distorted atomic arrangement from that found in space group *P*6/*mmm*. Curiously, both atomic arrangements with symmetry *P
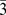
m*1 are currently known as the Cd_2_Ce-type.

**Table 1:** Crystal structure data of binary compounds in the Cd-Pr system; phase boundaries at 550°C are given according to Reichmann and Ipser [11].

Phase	Latticeparameter (Å)	Phase boundaries(at.% Cd)	Structuretype	Spacegroup	References
Cd_11_Pr	*a* = 9.287	91.67	BaHg_11_	*Pm-3m*	[16]
Cd_6_Pr	*a* = 15.689	85.71	Cd_6_Y	*Im-3*	[17]
Cd_58_Pr_13_	*a* = 15.71	80.76–81.79	Pu_13_Zn_58_	*P6_3_/mmc*	[18]
	*c* = 15.52				
Cd_45_Pr_11_	*a* = 21.842	79.98–80.36	Cd_45_Sm_11_	*F-43m*	[18]
Cd_3_Pr	*a* = 7.186	75.00–76.19	BiF_3_	*Fm-3m*	[16]
Cd_2_Pr	*a* = 5.025	65.80–66.67	Cd_2_Ce	*P-3m1*	[19]
	*c* = 3.459				
CdPr	*a* = 3.822	47.07–50.00	CsCl	*Pm-3m*	[16]

Single crystal X-ray diffraction was performed in the current study to determine an accurate *z* value for the Cd position in Cd_2_Pr, as described in chapter 3. Although Iandelli and Palenzona [19] did not find evidence for any solid solubility of Cd_2_Pr, Reichmann and Ipser [11] suggested a homogeneity range of around 1 at. %.

The compound Cd_3_Pr was first described by Iandelli and Ferro [16] to crystallize in the BiF_3_ structure type (*D*0_3_). Compounds with the composition ratio 3∶1 were found in most of the phase diagrams of Cd with lanthanides and appear with three different structure-types. In an early study of Bruzzone et al [18], who applied single crystal methods, the BiF_3_ structure was confirmed as to be the structure-type of Cd_3_Ce, Cd_3_Pr and Cd_3_Nd. This structure is coincident with a face centered cubic cell, formed by the respective rare earth element, where all cubic [8]-fold coordinated positions are occupied with Cd atoms [16].

In the very narrow composition range 79–82 at.% Cd, two intermetallic compounds are occurring: Cd_45_Pr_11_ (*cF*448) and Cd_58_Pr_13_ (*hP*142) [18]. Their unit cells are rather big, and compounds at these composition ratios appear irregularly in phase diagrams between Cd and lanthanides. Cd_45_Pr_11_ is isostructural with Cd_45_Sm_11_, which was investigated by single-crystal X-ray diffraction [26]. This structure type can be described by the so-called cluster concept originally adopted by Bradley and Jones [27]. Cd_45_Pr_11_ is thus an arrangement of 16 clusters of two types which are distributed along a NaTl-type unit cell. The two types of clusters which build up the Cd_45_Pr_11_ cell are related to clusters found in γ-brass phases and in α-Mn, respectively.

Cd_58_Pr_13_ forms a complex atomic arrangement that is isotypic to Cd_58_Ln_13_ where Ln stands for: La, Ce, Nd, Sm, Eu and Gd [18], [24], [28]–[30]. The basically hexagonal structure type contains various building blocks going along with extensive order-disorder phenomena resulting in distinct superstructures. Partly, an incommensurate behaviour is observed. Recently Piao et al. [31] discussed for the compound Ce_12.60_Cd_58.68_ a metrically commensurate representative which obviously represents a lock-in phase.

The compounds richest in Cd are Cd_6_Pr and Cd_11_Pr. Compounds with these composition ratios appear in all phase diagrams between Cd and lanthanides as line compounds, and Cd_6_Pr and Cd_11_Pr are no exceptions, as determined by Reichmann and Ipser [11]. Johnson et al. [17] presented cell parameters for a number of compounds with the composition ratio 6∶1. No additional information was given except that all homologues seem to be isomorphous. Subsequently, Larson and Cromer [32] investigated the crystal structure of Cd_6_Y using single-crystal X-ray diffraction. They reported that the crystal structure is isotypic to Ru_3_Be_17_ but with an additional Cd position in a 24-fold position and the site occupation factor of 0.33. Thus the structure-type of Cd_6_Pr is considered to be YCd_6_ (*cI*168). For the 1∶11 stoichiometry two modifications are known, namely BaCd_11_ (*tI*48) and BaHg_11_ (*cP*36), where Cd_11_Pr is crystallizing in the latter structure type.

Due to the limited information about phase relations and invariant reaction temperatures, it was the aim of the present work to provide an experimental investigation of the Cd-Pr phase diagram. In addition, these data can serve as input into a CALPHAD-type optimization of the Cd-Pr system. This would allow a better understanding of the distribution behaviour of Pr in the electro-refining cell described above.

## Experimental

All samples were prepared from pure elements, using Cd shot (99.9999%, AlfaAesar, Johnson Matthey Chemicals, Karlsruhe, Germany) and Pr pieces (99.9%, Smart Elements, Vienna, Austria). The surface-oxide layer of the Pr pieces was removed with a file. The elements were weighed with a semi-micro balance to an accuracy of about ±0.5 mg, resulting in an accuracy of ±0.01 at.% in the nominal composition for a total sample mass of 1 g. To prevent Pr from oxidation, the whole sample preparation was carried out in a glove box under Ar atmosphere (oxygen level: <1 ppm, water level: <1 ppm). The metals were placed in Ta crucibles which were designed in our laboratory and subsequently enclosed by means of arc welding under an Ar atmosphere of 0.25 bar.

For equilibration, the crucibles were sealed into silica glass tubes under a dynamic vacuum of better than 10^−2^ mbar. Afterwards, melting and annealing procedures were performed in muffle furnaces. The corresponding heat treatments are listed in Table 2. Samples were slowly heated (0.5 K/min) to temperatures above the melting point of Cd; this allows that Cd reacted with Pr rather completely before reaching elevated temperatures. It was realized that the heating step had a decisive influence on the quality of the final samples. Since Cd has a high vapour pressure above its melting point [33] and boils at 767°C, it builds up a rather high pressure inside the closed crucibles when present as pure metal. It was observed several times that samples with rather high Cd contents, heated too fast, showed a drastic expansion of the Ta crucibles. Sometimes, condensed Cd was found at the inner top of the Ta crucibles, resulting in a large deviation of the corresponding sample compositions. This behaviour could be avoided by a slow heating rate. To reach a homogeneous distribution of the elements, all samples with a Pr content higher than 10 at.%, were heated above the overall melting point of the respective alloys or at least above the melting point of Pr. All other samples which contained high amounts of Cd were heated to temperatures below the boiling point of Cd, to prevent condensation of Cd at the inner top of the crucibles, and cooled quickly by removing them from the furnace. Annealing and maximum temperatures are listed in Table 2. Quick cooling was important to obtain as-cast alloys for further investigations of the crystallisation behaviour but it helped also to obtain small grains which guaranteed short diffusion paths during any following annealing step. Samples were annealed for at least five weeks and subsequently quenched in cold water.

**Table 2:** Experimental phase compositions and lattice parameters of selected Cd-Pr samples.

Sample		Phase analysis		SEM	
/nom. comp.	Heat treatment	Phase	Latticeparameter (Å)	Cd (at.%)	Pr (at.%)
(at.%)	*T* (°C); duration; *T*_Max_ (°C)^c^				
1	300; 3 months; 350	Cd	too ductile	100	0.0
Cd_98_Pr_2_		Cd_11_Pr		91.5	8.5
2	300; 3 months; 350	Cd	too ductile	100	0.0
Cd_96_Pr_4_		Cd_11_Pr		91.5	8.5
3a	300; 2 months; 700	Cd	*a* = 2.9788(2),*c* = 5.6134(5)	100	0.0
Cd_93_Pr_7_		Cd_11_Pr	*a = *9.3027(2)	91.4	8.6
3b	300; 3 months; 700	Cd	*a* = 2.9812(5),*c* = 5.6165(2)	100	0.0
Cd_93_Pr_7_		Cd_11_Pr	*a = *9.3076(4)	91.5	8.5
4a	540; 2 months; 900	Cd_11_Pr	*a* = 9.3047(1)	91.5	8.5
Cd_88_Pr_12_		Cd_6_Pr	*a = *15.6904(2)	85.6	14.4
4b	540; 3 months; 700	Cd_11_Pr	*a* = 9.3046(3)	91.5	8.5
Cd_88_Pr_12_		Cd_6_Pr	*a = *15.6919(4)	85.9	14.1
5a	600; 2 months; 1050	Cd_6_Pr	*a* = 15.6933(2)	85.5	14.5
Cd_84_Pr_16_		Cd_58_Pr_13_	*a* = 15.6937(4),*c* = 15.5145(6)	81.8	18.2
5b	600; 3 months; 1050	Cd_6_Pr	*a* = 15.6953(6)	85.5	14.5
Cd_84_Pr_16_		Cd_58_Pr_13_	*a* = 15.6934(1),*c* = 15.5189(2)	81.8	18.2
6a	600; 2 months; 1050	Cd_58_Pr_13_	*a* = 15.7032(4),*c* = 15.4759(6)	80.8	19.2
Cd_81_Pr_19_		Cd_45_Pr_11_	*a = *21.8484(7)	80.3	19.7
6b	600; 3 months; 1050	Cd_58_Pr_13_	*a* = 15.6911(1),*c* = 15.4731(2)	80.9	19.1
Cd_81_Pr_19_		Cd_45_Pr_11_	*a = *21.8428(2)	80.0	20.0
7	800; 2 months; 800	Cd_58_Pr_13_	*a* = 15.6894(1),*c* = 15.4660(2)	80.4	19.6
Cd_78.5_Pr_21.5_		Cd_3_Pr	*a = *7.1819(6)	76.3	23.7
8	650; 2 months; 1050	Cd_45_Pr_11_	*a* = 21.8424(3)	79.6	20.4
Cd_78_Pr_22_		Cd_3_Pr	*a* = 7.1920(6)	76.2	23.8
9	700; 3 months; 1050	Cd_45_Pr_11_	*a* = 21.8448(6)	79.6	20.4
Cd_78_Pr_22_		Cd_3_Pr	*a* = 7.1893(2)	76.3	23.7
10^a^	545; 6 weeks	Cd_3_Pr	*a* = 7.2014(4)	75.3	24.7
Cd_74.9_Pr_25.1_		Cd_2_Pr	*a* = 5.0463(2),*c* = 3.4447(3)	– b	– b
11^a^	680; 6 weeks	Cd_3_Pr	–	75.7	24.3
Cd_74.7_Pr_25.3_		Cd_2_Pr	–	– b	– b
12	650; 2 months; 1050	Cd_3_Pr	*a* = 7.2029(9)	75.5	24.5
Cd_71_Pr_29_		Cd_2_Pr	*a* = 5.0461(9),*c* = 3.4451(1)	66.3	33.7
13	700; 3 months; 1050	Cd_3_Pr	*a* = 7.1979(4)	75.8	24.2
Cd_71_Pr_29_		Cd_2_Pr	*a* = 5.0451(5),*c* = 3.4455(5)	66.6	33.4
14	800; 2 months; 1050	Cd_2_Pr	*a* = 5.0446(3),*c* = 3.4452(3)	65.6	34.4
Cd_63_Pr_37_		CdPr	*a = *3.8356(4)	50.1	49.9
15	650; 2 months; 1050	Cd_2_Pr	*a* = 5.0439(5),*c* = 3.4453(5)	65.4	34.6
Cd_58_Pr_42_		CdPr	*a = *3.8340(6)	50.0	50.0
16	700; 3 months; 1050	Cd_2_Pr	*a* = 5.0404(8),*c* = 3.4434(6)	65.6	34.4
Cd_58_Pr_42_		CdPr	*a = *3.8349(9)	49.9	50.1
17	700; 3 months; 1050	CdPr	*a* = 3.8498(8)	47.1	52.9
Cd_46_Pr_54_		β-Pr	less intensity	22.1	77.9
18	600; 3 months; 1020	CdPr	too ductile	47.1	52.9
Cd_40_Pr_60_		β-Pr		20.0	80.0
19	400; 5 months; 1050	CdPr	too ductile	47.6	52.4
Cd_37_Pr_63_		α-Pr		– b	– b
20	530; 2 months; 1050	CdPr	too ductile	47.3	52.7
Cd_33_Pr_67_		β-Pr		18.7	81.3
21	700; 3 months; 1020	β-Pr	too ductile	14.9	85.1
Cd_15_Pr_85_					
22	500; 3 months; 1020	α-Pr	too ductile	3.5	96.5
Cd_10_Pr_90_		β-Pr		15.1	84.9
23	550; 3 months; 1020	α-Pr	too ductile	3.4	96.6
Cd_8_Pr_92_		β-Pr		12.2	87.8
24	600; 3 months; 1020	α-Pr	too ductile	3.1	96.9
Cd_6_Pr_94_		β-Pr		9.5	90.5
25	400; 5 months; 1050	CdPr	too ductile	– b	– b
Cd_3_Pr_97_		α-Pr		1.8	98.2

a Samples prepared with an isopiestic vapour pressure method, see Reichmann and Ipser [11].

b Microstructure of the respective phase was too fine to measure accurately with EDX.

c Highest temperature during sample preparation.

Phase identification and precise lattice parameters of the different phases were obtained by means of powder X-ray diffraction (powder-XRD) using a Bruker D8 Discover Series 2 powder diffractometer in Bragg-Brentano pseudo-focusing geometry employing CuKα radiation. Data were collected by either a LynxEye silicon strip detector (exposure time: 2 h) or a solid state detector (exposure time: 4 h). Additional measurements were performed on a Guinier-Huber camera 670 operating with CuKα_1_ radiation and an image plate detector (measurement period: 2 h, 10 detection loops). For powder-XRD measurements small pieces of each sample were powdered in a tungsten carbide mortar. Special sample holders with X-ray transparent lids were used to prevent the sample powders from oxidation. The corresponding XRD patterns were analyzed and refined by means of the TOPAS 3 software, applying the fundamental parameter approach for peak profile modeling.

For single-crystal structure determination, a Nonius four-circle diffractometer equipped with a 300 μm capillary-optics collimator, graphite monochromatized MoKα radiation and a CCD detector, was used. Unit-cell parameters were obtained by least-square refinements of all 2θ values of the registered reflections. Corrections of Lorentz, polarization and adsorption effects were made and structure refinement was done with the software SHELXL-97 [34]. To prevent the present samples from oxidation during the experiments, they were coated with vaseline and measured under a dry nitrogen atmosphere. All further instrumental information is given in chapter 3.

For investigations of the microstructures, selected samples were embedded in phenolic hot mounting resin and then ground and polished. Grinding was carried out with silicon carbide abrasive paper (mesh size: 400, 600, 800, 1000 and 1200) using water as fluid. Although Pr containing alloys are essentially sensitive to water, it was sufficient to keep the grinding steps as short as possible to prevent oxidation. Grinded samples were intermediately stored in cyclohexane. For fine polishing of the surface a water-free diamond suspension was taken with kerosene as fluid.

Metallographic analyses were carried out on a binocular reflected-light microscope (Zeiss Axiotech 100) featured with a bi-refringent prism for differential interference contrast (DIC) imaging and equipment for operation under polarized light. Quantitative examinations of the microstructures were performed on a Zeiss Supra 55 VP environmental scanning electron microscope (SEM) using pure elements as standard materials and Co for the energy calibration of the energy-dispersive X-ray (EDX) detector signal. A 120 μm aperture was used and an acceleration voltage of 20 kV was applied. For imaging of the microstructures, a back-scatter detector was employed. Final compositions were calculated by conventional ZAF matrix correction from the measured X-ray intensities. The composition of each phase was measured at ten or more spots in order to minimize statistical errors and to obtain more reliable results. An estimated error of about ±0.5 at.% was assumed for all measurements.

Differential thermal analysis (DTA) was carried out on a Netzsch DSC 404 F1 Pegasus using closed Ta crucibles with flat bottoms and a constant argon flow of 50 mL/min, respectively. Sapphire was used as the reference material. The temperature was measured with type-S (Pt/Pt10%Rh) thermocouples which were calibrated at the melting points of the high purity metals Sn, Zn, Ag and Cu. The samples weighed usually around 150–200 mg and were positioned in good thermal contact to the crucibles. Optionally, samples were powdered and pressed into pills to ensure good contact of the individual grains for better diffusion. Two heating- and cooling-curves were recorded for each sample using a heating rate of 5 Kmin^−1^.

## Results and Discussion

**Figure 1:**
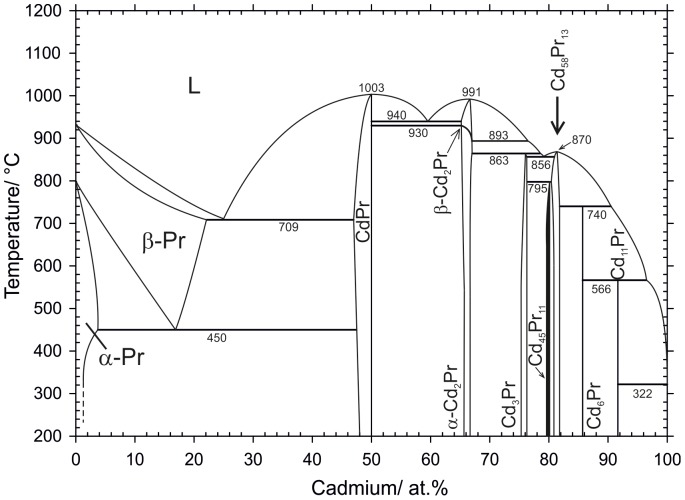
Cd-Pr phase diagram according to present results. The solubility of Pr in liquid Cd between 96.5–99.98 at.% Cd was taken from Johnson et al. [12].

A total number of 29 samples were annealed and characterized by powder-XRD, SEM and DTA to obtain a complete description of the Cd-Pr phase diagram. Equilibrated samples and as-cast alloys were consulted to define homogeneity ranges, phase equilibria and crystallization behaviour. All relevant samples, examined with isothermal methods, are listed in Table 2. Heat treatments, identified phases and phase compositions are given. The corresponding results are discussed in detail in chapter 1. Samples studied by DTA are listed in Table 3, together with their thermal effects from two heating and cooling cycles, respectively. These thermal effects are discussed properly in chapter 2. On the basis of the combined results a complete version of the Cd-Pr phase diagram was drawn, which is given in Fig. 1.

**Table 3:** Thermal effects of selected samples determined with DTA.

Sample	Nominalcomp. (at.%)	Annealingtemperature (°C)	Heating (°C)					Cooling (°C)	
			Invariant effects		Other effects			Liquidus	Liquidus
1	Cd_98_Pr_2_	300	323					558	520
2	Cd_96_Pr_4_	300	321	569				606	567
3	Cd_93_Pr_7_	300	322	567				690	669
4	Cd_88_Pr_12_	540	742	563				795	791
5	Cd_84_Pr_16_	600	738					851	838
6	Cd_81_Pr_19_	600	858					867	830
7	Cd_78.5_Pr_21.5_	800	856					865	860
8+9	Cd_78_Pr_22_	650 and 700	799	857				867	855
12+13	Cd_71_Pr_29_	650 and 700	865					968	948
14	Cd_63_Pr_37_	800	931	941				982	968
15+16	Cd_58_Pr_42_	650 and 700	928	939				960	956
17	Cd_46_Pr_54_	700						996	991
18	Cd_40_Pr_60_	600	456	709				953	951
19	Cd_37_Pr_63_	400	451	710				934	925
20	Cd_33_Pr_67_	530	449	704				886	873
21	Cd_15_Pr_85_	700	448		496		746	784	782
22	Cd_10_Pr_90_	700						832	834
23	Cd_8_Pr_92_	550			806			848	855
24	Cd_6_Pr_94_	600	450		670		820	870	871
25	Cd_3_Pr_97_	400			595	731	871	897	899
26	Cd_91.7_Pr_8.3_	500	566					708	687
27	Cd_85.7_Pr_14.3_	500	741					836	803
28	Cd_81.7_Pr_18.3_	500						870	838
29^a^	Cd_81.2_Pr_18.8_	552						870	863
30^a^	Cd_81.1_Pr_18.9_	558	859					869	842
31	Cd_80.4_Pr_19.6_	500	796	855				867	842
32	Cd_79.9_Pr_20.1_	750	791	854				866	849
33	Cd_75_Pr_25_	500	864					909	894
34	Cd_68_Pr_32_	800	860					986	980
35	Cd_67_Pr_33_	800	893					988	983
36	Cd_66.7_Pr_33.3_	500			909			991	959
37^a^	Cd_66.5_Pr_33.5_	632			915			991	986
38^a^	Cd_65.8_Pr_34.2_	732			923			986	984
39	Cd_55_Pr_45_	800	930	940				991	977
40	Cd_41_Pr_59_	400	454	702				959	958
41	Cd_29_Pr_71_	400	449	715				–	817
42	Cd_25_Pr_75_	400	448	711				711	–
43	Cd_21_Pr_79_	400	448		720			733	738
44	Cd_18_Pr_82_	400	446	565				755	752
45	Cd_12_Pr_88_	400	447		549		766	812	813
46	Cd_9_Pr_91_	400	453		608		796	838	843

a Samples prepared with an isopiestic vapour pressure method, see Reichmann and Ipser [11].

### 1. Results from Isothermal Methods

As can be seen in Table 2, several samples were prepared to determine the homogeneity ranges of the different intermetallic compounds at selected temperatures. All seven intermetallic compounds known from literature were confirmed with powder-XRD and SEM: Cd_11_Pr, Cd_6_Pr, Cd_58_Pr_13_, Cd_45_Pr_11_, Cd_3_Pr, Cd_2_Pr and CdPr. Estimated phase boundaries, defined at 550°C, fit quite well with values reported by Reichmann and Ipser [11] who applied an isopiestic vapour pressure method. Their values are included in Table 1 and indicate narrow but finite homogeneity ranges for all compounds except Cd_11_Pr and Cd_6_Pr, which were identified as line compounds also in the present study.

As seen from Table 2, not all samples could be examined with both methods, *i.e.* SEM and powder-XRD. Samples containing large amounts of Cd or Pr, respectively, turned out to be rather ductile; consequently powder-XRD could not be applied. Trials to grind two-phase samples of CdPr together with α/β-Pr into powders failed: apart from the fact that they were very ductile, Pr has a high atomic number and thus absorption effects were found to be quite high when irradiating with X-rays. The resulting low intensities made it impossible to perform an accurate evaluation of the lattice parameters. With respect to their ductility, no powder-XRD was carried out for samples 1 and 2 as well. Indeed, these results were assumed to be similar to the results from samples 3a and 3b, respectively.

The overall composition of each sample determined by SEM was compared with its nominal composition to estimate possible weight losses due to volatile Cd. No significant deviation of the determined composition from the nominal composition was found in most of the samples (*e.g.* sample 21 in Table 2). Even for samples with high Cd contents only a very thin Cd film could be observed visually inside the crucibles indicating negligible deviation from the nominal composition.

From the present results it was possible to derive the homogeneity ranges of the seven intermetallic compounds and of the solid solutions based on Pr and Cd. They are listed in Table 4. The phase boundaries on either side of Cd_11_Pr and Cd_6_Pr were found to deviate hardly at all from their corresponding stoichiometric compositions (*cf.* Table 2), *i.e.* the deviation was within the estimated error of the SEM measurements. Additionally, no significant variability of lattice parameters was observed, and thus Cd_11_Pr and Cd_6_Pr were treated as line compounds at their stoichiometric compositions.

**Table 4:** Maximum phase boundaries of Cd-Pr phases and of α–Pr and β–Pr (SEM) together with corresponding melting or decomposition temperatures averaged from DTA results.

Phase	Phase boundaries(at.% Cd)	Melting/decompositiontemperatures (°C)
Cd_11_Pr	91.7 (line compound)	566
Cd_6_Pr	85.7 (line compound)	740
Cd_58_Pr_13_	80.4–81.8	870
Cd_45_Pr_11_	79.6–80.4	795
Cd_3_Pr	75.0–76.3	863
Cd_2_Pr	65.2–67.0	991
CdPr	47.0–50.1	1003
β**–**Pr	0–22.1	–
α**–**Pr	0–3.6	–

**Figure 2:**
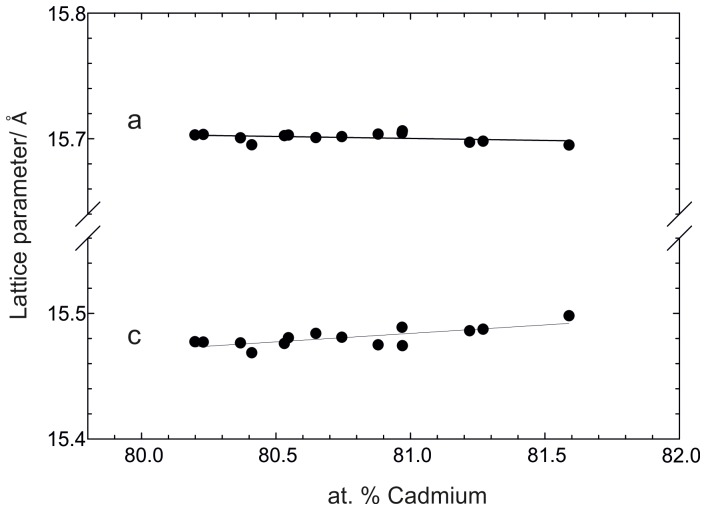
Lattice parameters *a* and *c* against at. % Cd within the homogeneity range of Cd_58_Pr_13_.

On the other hand, Cd_58_Pr_13_ exhibited clearly some solubility of Pr. As suggested by [11], the Pr solubility is temperature dependent. Its maximum solubility was predominantly described by sample 7 to be at around 80.4 at.% Cd at 800°C. Several single-phase samples of Cd_58_Pr_13_, taken from previous isopiestic vapour pressure experiments [11], were used to investigate lattice parameters. Although these samples were annealed at different temperatures between 480 and 600°C, phase compositions were plotted against cell parameters according to Vegard’s rule (Fig. 2). As can be seen, there is no significant change of lattice parameter *a* along the homogeneity range of Cd_58_Pr_13_ but with increasing Cd content the cell expands in the *c* direction. Since the covalent radius of Pr is definitely higher than the covalent radius of Cd one would expect the opposite behaviour. A possible explanation might be that in Cd_58_Pr_13_ vacancies on Cd sites are responsible for the deviation from stoichiometry to the Pr side. Thus, the higher the Cd content the lower would be the vacancy concentration which would explain the observed expansion. Furthermore, the formation of complicated superstructures has to be taken into account as found in the related Cd_58_Ce_13_ phase [31]. From samples 5a and 5b it can be seen that phase compositions at the Cd rich border of the homogeneity range of Cd_58_Pr_13_ were determined to be virtually identical to the stoichiometric composition, and thus the Cd solubility should be rather low.

**Figure 3:**
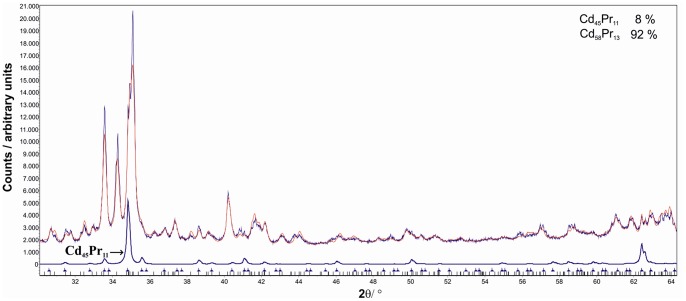
Powder-XRD pattern of an alloy with the nominal composition Cd_81_Pr_19_ (6a) which clearly contains both Cd_45_Pr_11_ and Cd_58_Pr_13_. ; red curve: calculated pattern.

Cd_58_Pr_13_ is in thermodynamic equilibrium with Cd_45_Pr_11_ within a very narrow two-phase field. In order to confirm this two-phase field, samples with the nominal composition Cd_81_Pr_19_ (samples 6a and 6b) were prepared and annealed at 600°C. Powder-XRD as well as SEM confirmed clearly the existence of the two-phase field Cd_58_Pr_13_+Cd_45_Pr_11_. The corresponding powder-XRD pattern of sample 6a is shown in Fig. 3. The latter sample was additionally used to determine the solubility of Cd in Cd_45_Pr_11_ at 600°C. The phase boundary was measured at 80.3 at.% Cd which corresponds exactly with the stoichiometric composition of Cd_45_Pr_11_. Three samples with the nominal compositions Cd_78_Pr_22_ (samples 8 and 9) and Cd_78.5_Pr_21.5_ (sample 7), respectively, were prepared and annealed at temperatures between 650 and 800°C to investigate a possible variation of the Pr-rich phase boundary of Cd_45_Pr_11_ with temperature. The phase boundary of Cd_45_Pr_11_ turned out to be the same at 650 and 700°C, both determined at 79.6 at.% Cd. However, when investigating sample 7, which had been annealed at 800°C, a two-phase equilibrium between Cd_58_Pr_13_ and Cd_3_Pr was observed; the expected phase Cd_45_Pr_11_ was absent. Thus Cd_45_Pr_11_ must decompose at some temperature lower than 800°C. The corresponding DTA results are given in detail in section 2.

Although [11] described that Cd_3_Pr is only slightly more stable than a two-phase mixture of its neighbouring phases, in the present study it was found to form reliably as an intermediate compound between Cd_45_Pr_11_ and Cd_2_Pr. Seven samples (7–13) were consulted to describe the phase boundaries of Cd_3_Pr accurately between 545 and 800°C. As can be seen in Fig. 1 the homogeneity range is shifted towards compositions richer in Cd. Obviously, the homogeneity range does not include the stoichiometric composition at elevated temperatures. Nevertheless, the Pr-rich phase boundary of Cd_3_Pr reaches 75.3 at.% Cd at 545°C. This value is indeed close to the stoichiometric composition, which would be reached by an extrapolation to lower temperatures.

**Figure 4:**
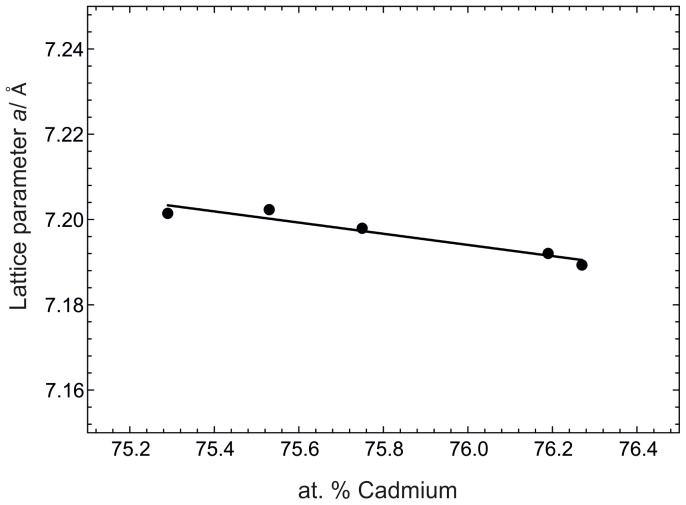
Lattice parameter *a* against at.% Cd within the homogeneity range of Cd_3_Pr.

This relatively broad homogeneity range suggested an investigation of the defect mechanism. Therefore, the lattice parameter *a* was plotted *vs.* the composition in Fig. 4. This plot is based on results from samples 8–11 and 13. A decrease of the lattice parameter *a* with increasing Cd content is indicated which is in agreement with the smaller covalent radius of Cd. Since interstitial Cd sites are unlikely within this close packed BiF_3_-type structure, there are either mixed Cd/Pr positions or vacancies formed at the Pr site. To learn more about the defect mechanism, single-crystal X-ray diffraction was performed and the results are discussed in 3.

From the present results a homogeneity range of 65.4–66.7 at.% Cd could be estimated for Cd_2_Pr at 550°C which is in good agreement with values given by [11]. No significant change of the lattice parameters was observed with varying composition. When grinding samples containing Cd_2_Pr and CdPr, it appeared that these compounds are rather ductile. Vickers hardness measurements of single-phase Cd-Gd alloys were carried out by Bruzzone et al. [35]. When comparing the values of the different Cd-Gd compounds, one can recognize that especially the phases Cd_2_Gd and CdGd show relatively small values. Since Cd_2_Gd and CdGd are isotypic with the present phases Cd_2_Pr and CdPr a similar mechanical behaviour was expected; in fact, it was extremely difficult to powder these samples. Consequently, the XRD-powder patterns of Cd_2_Pr and CdPr showed rather broad peak shapes. Additionally, absorption phenomena of these Pr-rich phases lowered also the intensities and made it altogether difficult to derive accurate lattice parameters.

**Figure 5:**
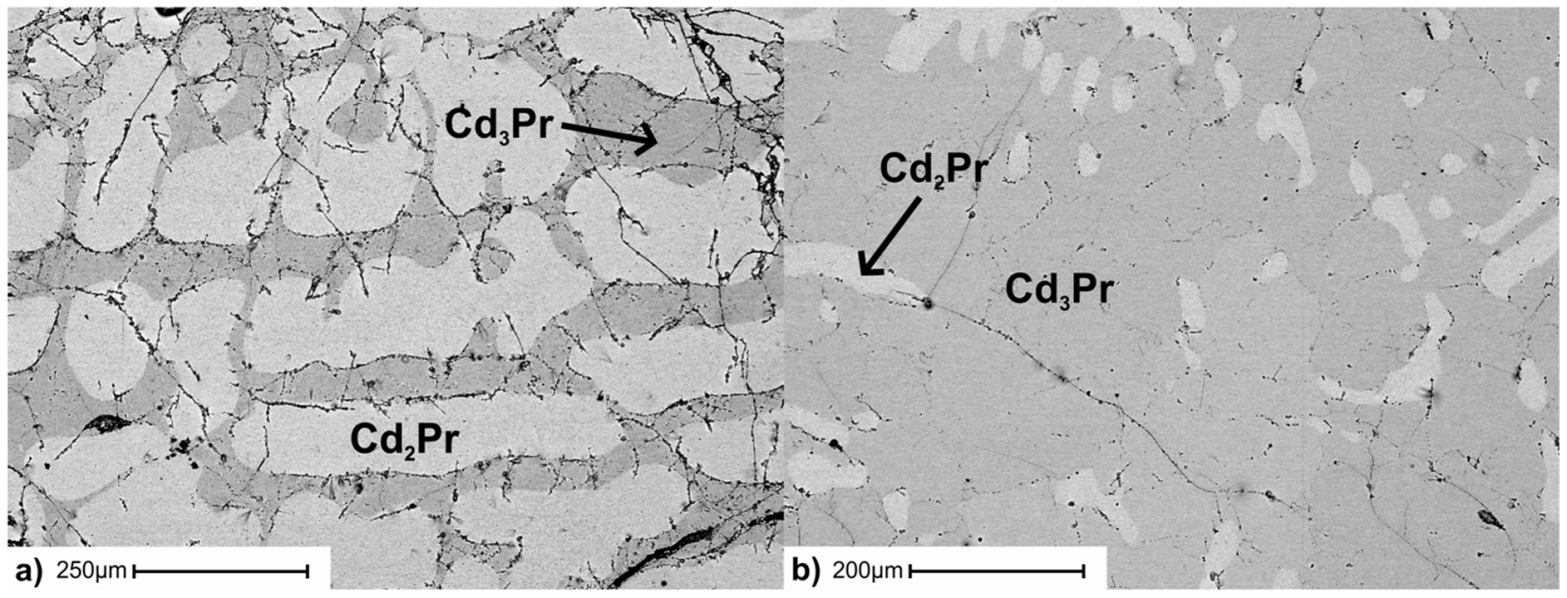
BSE image of an a) as-cast alloy with the stoichiometric composition of Cd_2_Pr and b) as-cast alloy with the stoichiometric composition of Cd_3_Pr.

For structural investigations suitable single-crystals were obtained from an as-cast alloy with the stoichiometric composition Cd_2_Pr. The corresponding sample was analysed with SEM to consist of Cd_2_Pr and Cd_3_Pr grains. Phase compositions were found to be Cd_66.2_Pr_33.8_ (Cd_2_Pr) and Cd_75.8_Pr_24.2_ (Cd_3_Pr), respectively. Its microstructure exhibited clearly a primary crystallization of Cd_2_Pr (*cf.* Fig. 5a). The results of a single crystal X-ray study of this phase can be found in chapter 3.

**Figure 6:**
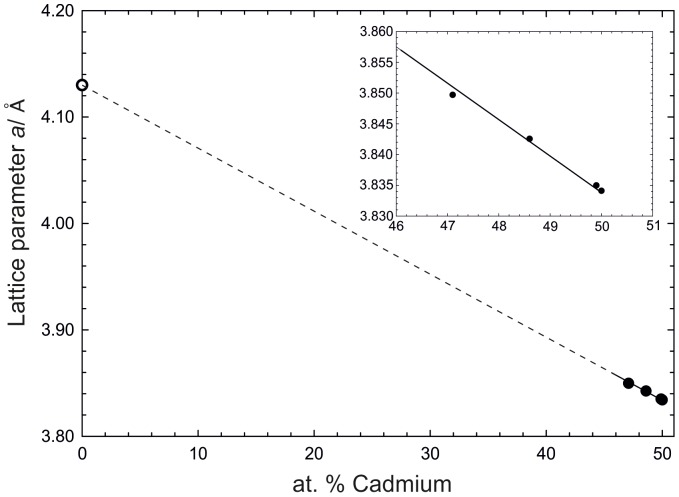
Lattice parameter *a* against at.% Cd for CdPr. ; full circles: sample compositions defined by SEM (see text).

The compound richest in Pr is CdPr. It crystallizes in the CsCl-structure type and exhibits an ordered variety of the β-Pr structure which crystallizes in the *bcc* W-structure (*a = *4.1300 Å [36]); half of the Pr sites in β-Pr are substituted by Cd atoms. Besides, a partial substitution of Pr by Cd according to Cd_1−*x*_Pr_1+*x*_, *x* <0.06, was observed. The two-phase field β-Pr+CdPr can be considered as a large miscibility gap which cuts off a continuous solid solution (with a second-order phase transition) between these two phases. Samples with major amounts of Cd_1−*x*_Pr_1+*x*_, were used to show the correlation of the lattice parameter *a* with the composition (Fig. 6). The increasing Pr content goes along with an increase of the lattice parameter which is in perfect agreement with the relative atomic radii of Pr and Cd; the excellent linear fit (Fig. 6) corresponds with Vegard’s rule.

### 2. Results of Thermal Analyses

**Figure 7:**
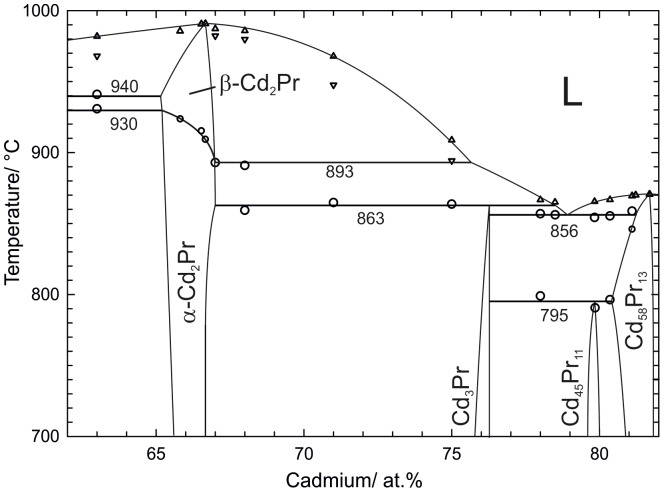
Partial phase diagram of Cd-Pr between 61 and 82 at.% Cd. Large circles: invariant thermal effects. Small circles: non-invariant effects. Triangles up: liquidus on heating. Triangles down: liquidus on cooling.

Binary phase reactions were examined by means of DTA. For the description of the reaction scheme and the graphical representation of the phase diagram (Fig. 1) samples listed in Table 2 as well as additional equilibrated samples and such from isopiestic experiments [11] were used. Effects measured from samples with equal nominal compositions were averaged. The corresponding results of the DTA measurements are given in detail in Table 3. For each sample two heating and cooling cycles were performed at a heating rate of 5 K/min in order to check if equilibrium conditions can be restored after the first melting of the sample. It was found that most of the specimens were still in equilibrium after the first cycle. Nevertheless, for the graphical representation of the phase diagram all thermal effects, except the liquidus, were taken from the first heating curves. In case of the liquidus effect, usually an average value from the two heating curves was taken for the evaluation. Although most of the cooling curves exhibited considerable supercooling, their liquidus values were still considered to guide the construction of the liquidus curves shown in Figs. 1 and 7.

All invariant reactions, evaluated from the present results, are listed in Table 5 together with the respective reaction temperatures and types and the phase compositions. As can be seen, the intermetallic compounds Cd_11_Pr, Cd_6_Pr, Cd_45_Pr_11_ and Cd_3_Pr are formed incongruently whereas Cd_58_Pr_13_, Cd_2_Pr and CdPr show congruent melting behaviour. For measuring the melting or decomposition temperatures of these compounds pure samples were required. Therefore, alloys with stoichiometric compositions were subjected to special treatments. They were heated within 24 hours to a final temperature of 750°C, i.e., slightly below the boiling point of Cd, and held for 48 hours to reach a homogeneous distribution of the elements. Afterwards the temperature was slowly reduced to room temperature. All alloys were checked by powder-XRD to consist of single-phases only; the corresponding melting or decomposition temperatures are included in Table 4.

**Table 5:** Invariant reactions in the system Cd-Pr derived from a combination of all present results.

Reaction	*T*/°C	Phase compositions (at.% Cd)			Reaction type
L+Cd_11_Pr 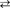 Cd	322±2	∼100	91.7	∼100	degenerate peritectic
L+Cd_6_Pr 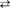 Cd_11_Pr	566±3	96.5^a^	85.7	91.7	peritectic
L+Cd_58_Pr_13_ 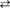 Cd_6_Pr	740±3	90.5	81.8	85.7	peritectic
Cd_58_Pr_13_ 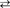 L	870±2		81.7		congruent melting
Cd_58_Pr_13_+ Cd_3_Pr 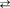 Cd_45_Pr_11_	795±5	80.4	76.3	79.8	peritectoid
L 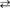 Cd_58_Pr_13_+ Cd_3_Pr	856±3	78.9	81.3	76.3	eutectic
Cd_2_Pr 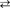 L	991±2		66.7		congruent melting
L+α-Cd_2_Pr 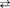 Cd_3_Pr	863±3	78.5	67.0	76.3	peritectic
α-Cd_2_Pr 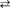 β-Cd_2_Pr	893±4		67.0		polymorphic transformation
	930±2		65.2		
CdPr 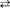 L	1003±2		50.0		congruent melting
L 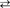 β-Cd_2_Pr+CdPr	940±2	59.1	65.2	50.1	eutectic
L 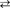 CdPr+β-Pr	709±5	25.0	47.0	22.1	eutectic
β-Pr 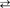 CdPr+α-Pr	450±5	16.8	47.1	4.0	eutectoid

a value was taken from Johnson et al. [12].

At the very Cd-rich side of the phase diagram a degenerate isothermal reaction takes place which could be either a eutectic or a peritectic reaction, respectively. Samples with nominal compositions within the two-phase field Cd+Cd_11_Pr were studied by DTA to define the reaction temperature. Unfortunately, no significant evidence could be obtained for either case because the melting point of Cd is very close to the reaction temperature. Therefore, a DTA investigation of a sample with the nominal composition Cd_96_Pr_4_ (sample 2, Table 3) was performed against pure Cd as a reference material. Assuming, that both sample and reference position are symmetrical within the furnace, one would expect an effect at first caused by the sample if the respective reaction exhibits a degenerate eutectic. Contrary, one would expect to observe first the melting point of Cd if a degenerate peritectic reaction takes place. Since Cd is used as the reference material, its melting effect is hypothetically exothermal and thus in the opposite direction in the DTA curve, compared to effects from the samples. From the present experiment it was clearly found that Cd melted first. Thus a degenerate peritectic reaction was considered for the formation of Cd.

The two line-compounds Cd_11_Pr and Cd_6_Pr decompose peritectically at 566 and 740°C, respectively. The peritectic reaction temperature of Cd_11_Pr fits excellently with the value of 570°C, reported by Johnson et al [12]. Although there were no experimental data available, Kurata and Sakamura [15] calculated an isothermal reaction temperature of 678°C for the peritectic formation of Cd_6_Pr. As can be seen this value is quite different from the presently obtained one at 740°C.

The liquidus between 100 and 96 at.% Cd is rising rather steeply (Fig. 1). Its shape was drawn according to results from Johnson et al. [12] which agree quite well with the present results. Johnson et al. [12] determined the solubility of Pr in liquid Cd by chemical analysis of filtered samples. From an empirical fit of their solubility data the authors calculated that 3.5 at.% Pr are soluble in liquid Cd at 570°C. This solubility was considered in the present version of the phase diagram to be the liquidus composition at the peritectic formation temperature of Cd_11_Pr.

In the narrow composition range 62 to 82 at.% Cd four intermetallic compounds are located and several invariant reactions take place (see Fig. 7). As described above, the two compounds Cd_58_Pr_13_ and Cd_45_Pr_11_ are in thermodynamic equilibrium within a very narrow two-phase field and the decomposition of Cd_45_Pr_11_ must occur at some temperature below 800°C. A rather weak effect was found in samples containing Cd_45_Pr_11_ and the corresponding average value was 795°C. Due to the fact that these effects were rather weak and that no Cd_45_Pr_11_ was found at 800°C it was reasonable to assume a solid-phase reaction, *i.e.* a peritectoid formation of Cd_45_Pr_11_.

**Figure 8:**
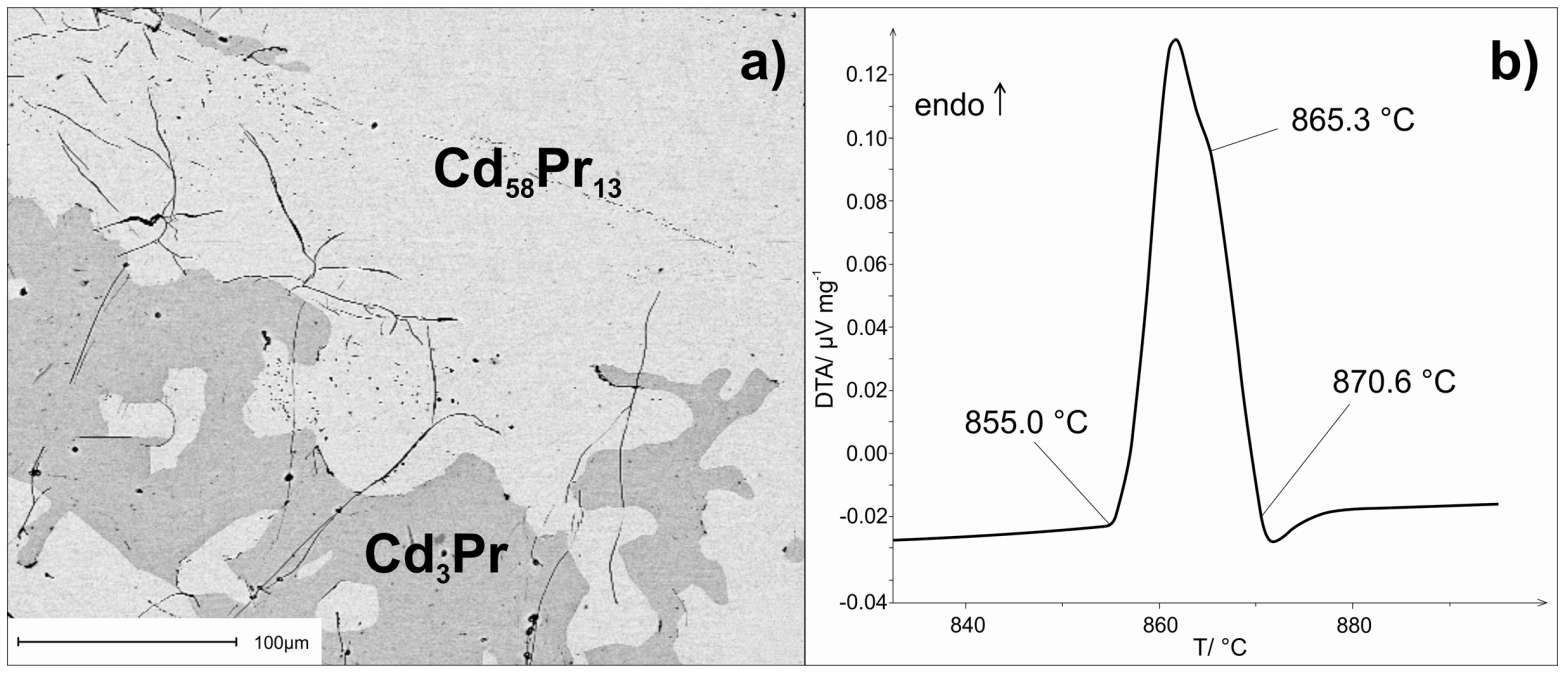
a) Microstructure of an as-cast alloy with the stoichiometric composition of Cd_45_Pr_11_; b) DTA curve of sample 7 against pure Cd_58_Pr_13_ as reference.

To determine the primary crystallization behaviour of as-cast alloys at the stoichiometric compositions Cd_58_Pr_13_ and Cd_45_Pr_11_, their microstructures were investigated. The respective microstructure of an as-cast alloy with the stoichiometric composition of Cd_45_Pr_11_ is shown in Fig. 8a. As Cd_58_Pr_13_ is the primary crystallizing phase and Cd_3_Pr forms the matrix, a eutectic reaction was assumed between these two phases; this was also indicated from the DTA experiments that provided an average eutectic temperature of 856°C. To confirm the congruent formation of Cd_58_Pr_13_, a sample with the nominal composition Cd_78.5_Pr_21.5_ was annealed at 800°C (sample 7); pure Cd_58_Pr_13_ served as the reference material for the DTA. The procedure was exactly the same as described above. The corresponding DTA curve is given in Fig. 8b. As expected, there was at first an endothermic effect in the sample, dedicated to the eutectic reaction, followed by the liquidus of the sample, measured at 865.3°C. Subsequently, the melting effect of Cd_58_Pr_13_ appeared at around 870.6°C in terms of a hypothetical exothermal effect. Thus, the congruent formation of Cd_58_Pr_13_ is apparent.

When investigating samples located in the two-phase field (CdPr+Cd_2_Pr) two effects were found to occur at 930 and 940°C, respectively. Clearly, there is a eutectic reaction between the congruently melting compounds CdPr and Cd_2_Pr at 940°C. The eutectic point could be located between 58.0 and 59.4 at.% Cd. As-cast alloys at these compositions showed primary crystallization of CdPr and Cd_2_Pr, respectively. However, the effect at lower temperature was unexpected. Single-phase samples of Cd_2_Pr exhibited small effects slightly below 930°C. Thus a structural transformation of Cd_2_Pr was assumed similar to the situation reported for Cd_2_Gd [35] or Cd_2_Dy [19], [20]. DTA results of samples, located in the homogeneity range as well as adjacent two-phase fields of Cd_2_Pr, showed that the polymorphic transformation is temperature dependent and varies between 893°C on the Cd-rich side and 930°C on the Pr-rich side. Several attempts to quench the high temperature modification β-Cd_2_Pr were not successful but attempts to clarify its structure are still continuing.

Several samples were investigated to clarify the homogeneity range of the high-temperature modification of Pr exactly. Respective DTA results fit excellently with phase compositions obtained from EDX measurements. Therefore, the maximum solubility of Cd in β-Pr could be determined reliably and is given as around 22.1 at.% Cd (*cf.* sample 17). From the determined liquidus temperatures of all samples located within 0–50 at.% Cd, the eutectic point L
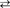
CdPr+β-Pr was estimated to be around 25 at.% Cd. From DTA of twelve samples the eutectoid decomposition temperature of β-Pr was averaged and a value of 450°C was obtained. Veleckis and Van Deventer [14] listed isothermal reaction temperatures for eutectic reactions between lanthanides and the respective compound richest in lanthanide, which is, with the exception of Cd_5_Eu_6_, CsCl-structured CdLn (Ln = lanthanide). As indicated by Gschneidner and Calderwood [13] the eutectic temperatures do not fit well with subsequently reported values. The largest differences between values given by Veleckis and Van Deventer and those reported later occurred when the corresponding lanthanide undergoes an allotropic transformation. The present authors assume that Veleckis and Van Deventer did not consider the high-temperature allotropic modifications of the respective lanthanides. Indeed, their reported value for the eutectic formation of Pr and CdPr, given as 435°C, corresponds quite well with the present value for the eutectoid decomposition of β-Pr.

### 3. Single-crystal X-ray Study of Cd_3_Pr and Cd_2_Pr

Fig. 4 shows a decrease of the lattice parameter *a* with increasing Cd content in Cd_3_Pr. This behaviour is following Vegard’s rule and is expected when considering that the covalent radius of Cd is noticeably smaller. As described above the most probable defects are mixed positions or vacancies on the Pr site of the Cd_3_Pr lattice.

Single-crystal X-ray diffraction experiments were performed in the present study to determine the defect mechanism in Cd_3_Pr accurately. Suitable single crystals were found in an as-cast alloy with the stoichiometric composition of Cd_3_Pr. The corresponding microstructure is shown in Fig. 5b. The atomic arrangement was refined from single-crystal X-ray diffraction data (see Table 6). Atomic coordinates, occupation factors and equivalent isotropic displacement parameters are listed in Table 7. The structure of Cd_3_Pr consists of three atomic positions, all originally described as special positions. Pr atoms are located at the site 4(*a*) exhibiting minor vacancies. The Cd atoms are located at the fully occupied site 4(*b*) and the site 32(*f*), roughly occupied to a quarter. Attempts to refine the crystal structure in any subgroup of *Fm*−3*m* to establish an ordered structure model failed. Considering vacancies at the Pr and Cd(2) sites reduced the R-values significantly.

**Table 6:** Details for single-crystal X-ray data collections and structure refinements for the compounds Cd_2_Pr and Cd_3_Pr.

Compound	Cd_2_Pr	Cd_76.4_Pr_23.6_
Crystal system, space group	hexagonal, *P6*/*mmm*	cubic, *Fm 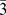 m*
*a*/Å	5.043 (2)	7.204 (1)
*c*/Å	3.455 (1)	–
ρ_calc/_g•cm^−3^; μ(*MoKα*)/mm^−1^	7.98; 29	8.49; 29
Z	1	4
Total reflections measured	1022^a^^,^ *	1443^a^^,^ **
Unique reflections (*n*); reflections with *F_o_ >4σ*(*F_o_*)	87; 82	63; 58
*R_int_* = *Σ |F_o_^2^*−*F_o_^2^*(mean)*|/Σ F_o_^2^*	0.0459	0.0272
*R*1 = *Σ* (*||F_o_|−|F_c_||*)*/Σ |F_o_|* observed; all reflections	0.0299; 0.0319	0.0168; 0.0178
*w*R^2^ = [*Σ w*(*F_o_^2^*−*F_c_^2^*)*^2^/Σ wF_o_^4^*]*^1/2^*	0.0720	0.0371
GooF = [*Σ w*(*F_o_^2^*−*F_c_^2^*)*^2^/*(*n-p*)]*^1/2^*	1.187	1.093
Extinction parameter	0.016(14)	0.0019(2)
Max Δ/σ; number of variable parameters (p)	<0.001; 6	<0.001; 9
Final difference Fourier map/eÅ^−3^	−2.02 to +2.25	−0.71 to +0.97

a NONIUS four-circle diffractometer (equipped with a CCD detector and a 300 μm capillary-optics collimator, Mo tube, graphite monochromator; 30 mm crystal-detector distance; rotation angle 2° per image, φ-scans at 14 distinct ω-angles). Structure refinements were performed with program SHELXL (Sheldrick [34]).

*513 measured frames, exposure time of 145 seconds per degree.

**514 measured frames, exposure time of 165 seconds per degree.

**Table 7:** Atomic coordinates and equivalent isotropic displacement parameters for Cd_2_Pr and Cd_3_Pr.

AtomicPosition	Wykoffletter	Occupationfactor	Atomic coordinates	*U_eq_*
**Cd_2_Pr**				
Pr	1(*a*)	1.0	(0 0 0)	0.0170(3)
Cd	2(*d)*	1.0	(⅔⅓½)	0.0199(3)
**Cd_3_Pr**				
Pr	4*a*	0.93(1)	(0 0 0)	0.0151(4)
Cd1	4*b*	1	(½½½)	0.0173(5)
Cd2	32*f*	0.246(2)	(*x x x*) *x* = 0.2392(5)	0.0297(10)

Moreover, the vacancies observed at the Pr site are in agreement with the analytically observed homogeneity range of Cd_3_Pr. The chemical composition calculated from the structure refinement (Cd_76.3_Pr_23.7_) is in excellent agreement with the composition detected by SEM investigations (Cd_75.9_Pr_24.1_).

Iandelli and Palenzona [19] as well as Mulokozi [22] described Cd_2_Pr to crystallize in the Cd_2_Ce-type, a deformed AlB_2_-type structure type with space-group symmetry *P
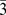
m*1, where Cd occupies the site 2(*d*) (⅓⅔ *z*) with z = 0.42. During the present work a suitable crystal of Cd_2_Pr with the dimensions 0.02×0.02×0.10 mm was used for single-crystal X-ray diffraction measurements. The corresponding data collection is given in Table 6. Atomic coordinates, occupation factors and equivalent isotropic displacement parameters are listed in Table 7. The structural refinement exhibited space-group symmetry *P*6*/mmm* with the Cd atom located at (⅓⅔½). Any attempts to refine the crystal structure with a lower symmetry failed. Consequently, Cd_2_Pr is also assumed to adopt the simple AlB_2_-type.

## Conclusion

The complete Cd-Pr phase diagram was investigated with a combination of powder-XRD, SEM and DTA; it is presented in Fig. 1. Based on the present results, the homogeneity ranges of the altogether seven intermetallic compounds and of the solid solution of Cd in Pr were derived (Table 4). The solubility ranges fit quite well with values reported previously by Reichmann and Ipser [11]. Additionally, melting or decomposition temperatures of all intermetallic compounds, derived from DTA measurements, are listed. It was found that the intermetallic compounds Cd_11_Pr, Cd_6_Pr, Cd_45_Pr_11_ and Cd_3_Pr are formed incongruently whereas Cd_58_Pr_13_, Cd_2_Pr and CdPr exhibit congruent formation behaviour. The invariant reaction temperature for the peritectic formation of Cd_11_Pr was comparable with values reported by Refs. [12] and [15]. The solid solubility of Cd in β-Pr could be determined reliably and is given as about 22.1 at.% Cd. The addition of Cd stabilizes the high-temperature modification β-Pr down to 450°C where it decomposes in terms of a eutectoid reaction.

A phase transformation of Cd_2_Pr is suggested by the DTA results. Several attempts to synthesize and quench the high temperature modification β-Cd_2_Pr were not successful but attempts are continuing to clarify its structure. The low-temperature modification of Cd_2_Pr was examined by single-crystal XRD and determined to crystallize in the simple AlB_2_-type. Furthermore, single-crystal X-ray diffraction was employed to explain the defect mechanism in Cd_3_Pr. Due to reduction in symmetry of one Cd position and the resulting introduction of ordered vacancies the R-values could be decreased noticeable.
